# A new species of *Knodus* Eigenmann (Characiformes: Characidae: Stevardiinae) with comments on nuptial tubercles and gill gland in characiform fishes

**DOI:** 10.1371/journal.pone.0217915

**Published:** 2019-07-10

**Authors:** Naércio A. Menezes, Manoela M. F. Marinho

**Affiliations:** Seção de Peixes, Museu de Zoologia da Universidade de São Paulo, São Paulo, São Paulo, Brazil; Pontificia Universidade Catolica do Rio Grande do Sul, BRAZIL

## Abstract

*Knodus nuptialis* n. sp. is described from the Rio Curuá drainage, Rio Xingu basin, Brazil. It can be diagnosed from its congeners by having dentary teeth decreasing gradually in size posteriorly, outer premaxillary teeth row with five cusps, 12–15 branched anal-fin rays and a single humeral spot. The species presents notable sexual dimorphism consisting of densely concentrated nuptial tubercles on head, body, and fins, gill-gland, and bony hooks in the anal fin of mature males. It was found that these sexually dimorphic features are useful and functional in males of the new species only during the reproductive season and after this period, they become atrophied, and eventually disappear. The list of characiform species presenting breeding tubercles is updated and nine species and two genera of the Characidae, *Deuterodon* and *Bryconacidnus*, are for the first time reported to have breeding tubercles.

## Introduction

The combination of two premaxillary tooth rows, inner row with four teeth, and caudal-fin scaled has been traditionally used to diagnose the characid genus *Knodus* Eigenmann, defined as being “a *Bryconamericus* in all but is scaled caudal” [1]. The recognition of *Knodus* has long been controversial, some consider it as synonym of *Bryconamericus* Eigenmann in Eigenmann (see discussion in [2–4]). In recent phylogenetic analyzes there is consensus that *Knodus* is not a natural assemblage as traditionally defined [5–7], and [6] further defined a “*Knodus sensu stricto*” based on molecular data, encompassing species of *Knodus*, *Bryconamericus* and *Bryconadenos*. Therefore, the genus still lacks a phylogenetic definition.

The species of *Knodus* are especially abundant in headwater streams and rivers with sand beaches flowing into the Amazon basin in central Brazil. Recent collecting expeditions undertaken to one of these areas in the Rio Curuá, a tributary of the Rio Xingu basin, provided a new species described herein. One of the samples is represented by fully mature males and females in which breeding tubercles, nodular structures widespread over the skin, and a gill gland, both found in many groups of fishes including representatives of the order Characiformes, presented us the opportunity to review, discuss, and add new information on them. The new species is described in *Knodus* following the traditional definition of the genus, pending further phylogenetic studies to better establish its relationships within characids.

## Material and methods

Counts and measurements are those described in [8] and [9] except for the number of longitudinal scale rows below the lateral line, which are counted from the pelvic-fin origin to the lateral line. The number of scale rows below lateral line includes the smaller and notched scale at the base of the lateralmost pelvic-fin ray. The pattern of radii was examined on scales sampled from the third horizontal scale row from dorsal-fin base to the lateral line. Numbers of vertebrae and vertebral elements, supraneurals, procurrent caudal-fin rays, teeth cusps and unbranched anal-fin rays were obtained from five cleared and stained (CS) specimens prepared according to [10]. In the list of specimens, mol indicate molecular tissue preserved. Vertebral counts include the four vertebrae of the Weberian apparatus as well as the first pre-ural and first ural centrum of the caudal region, counted as single element. In the description, the range of meristic data are followed by the frequency for each count in parenthesis, with an asterisk (*) indicating the value of the holotype. Institutional abbreviations follow [11]. Analyses for differences between sexes were performed using T-Test Calculator for 2 Independent Means in [12] using measurements not transformed into body proportions. Difference was considered significant when *p ≤* 0.05. Correlation of sexual dimorphic traits were confirmed upon examination of the gonads of most of the specimens, under the stereomicroscope. Color in life was based on photographs of freshly collected specimens.

Most specimens analyzed are from fish collections specified below. The specimens captured in this study were collected under permit number 26281–1 issued by the Instituto Brasileiro do Meio Ambiente e dos Recursos Naturais Renováveis (IBAMA), and the field studies did not involve endangered or protected species. They were euthanized with an overdose of anaesthetic MS-222 and then fixed in formalin. This study is part of the project number 226/2015 approved by Brazilian ethics committee Comissão de Ética no Uso de Animais (CEUA) do Instituto de Biociências da Universidade de São Paulo under Credenciamento Institucional para Atividades com Animais em Ensino ou Pesquisa Científica (CIAEP), number 01.0165.2014, Conselho Nacional de Controle de Experimentação Animal (CONCEA) do Ministério da Ciência, Tecnologia e Inovação (MCTI).

Data of other *Knodus* species were primarily obtained from the revision of the genus undertaken by [5], unless otherwise stated. For those species described after 2007, the data were taken from their original descriptions.

### Nomenclatural acts

The electronic edition of this article conforms to the requirements of the amended International Code of Zoological Nomenclature, and hence the new names contained herein are available under that Code from the electronic edition of this article. This published work and the nomenclatural acts it contains have been registered in ZooBank, the online registration system for the ICZN. The ZooBank LSIDs (Life Science Identifiers) can be resolved and the associated information viewed through any standard web browser by appending the LSID to the prefix “http://zoobank.org/”. The LSID for this publication is: urn:lsid:zoobank.org:pub: 5600BE6F-2459-4901-BC66-F5D37F0D6679. The electronic edition of this work was published in a journal with an ISSN, and has been archived and is available from the following digital repositories: PubMed Central, LOCKSS.

## Results

### *Knodus nuptialis*, new species

urn:lsid:zoobank.org:act:31DC2F83-99F6-4EC6-A250-A196C90AB36F

Figs 1–8, Table 1

**Fig 1:**
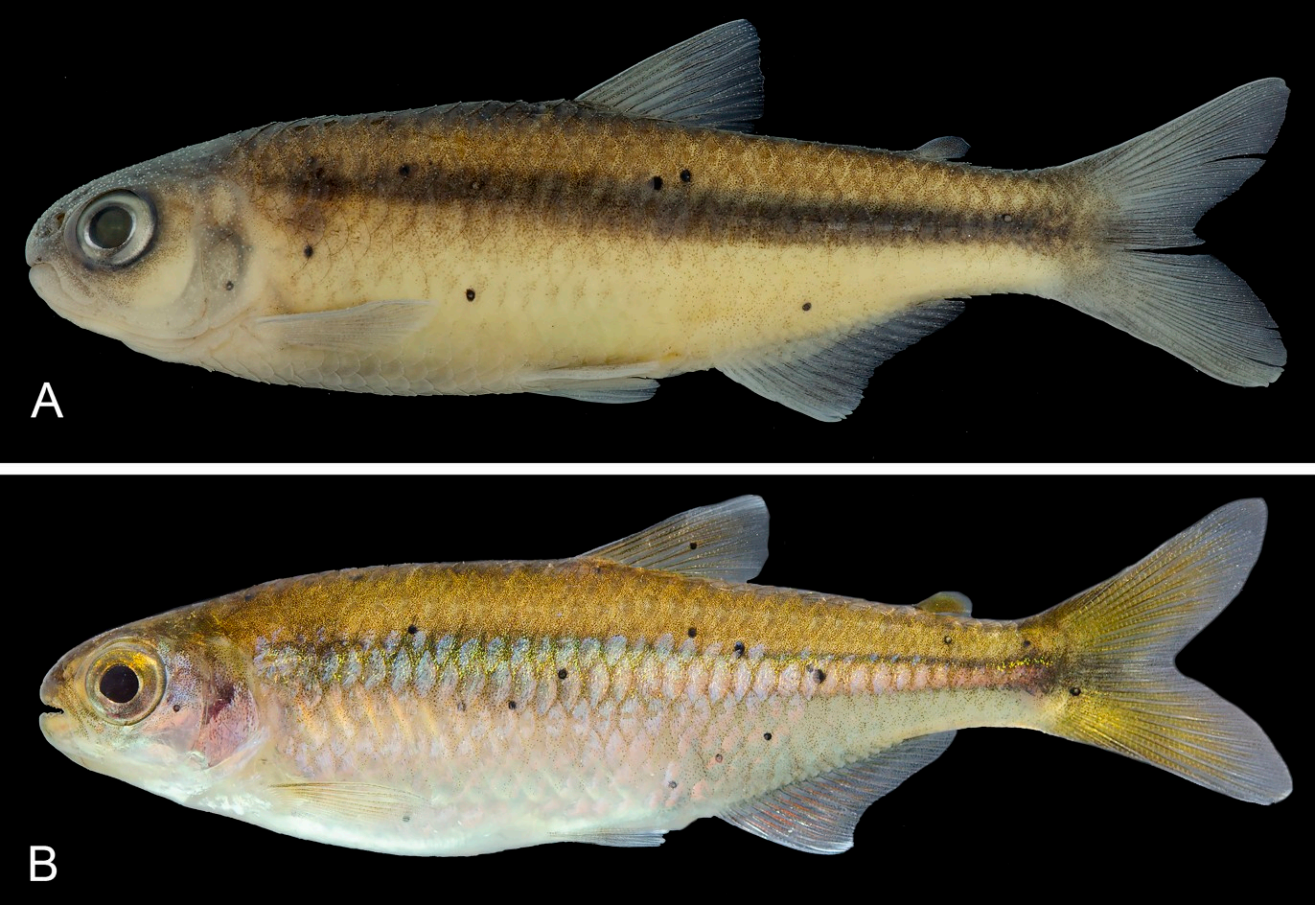
Type specimens of *Knodus nuptialis*. Brazil, Pará, Altamira, Rio 13 de Maio at PCH Salto do Três de Maio, tributary of Rio Curuá, Rio Xingu basin (A) holotype, preserved coloration, MZUSP 124829, 46.5 mm SL, male; (B) paratype MZUSP 124828, female, 50.8 mm SL.

**Fig 2:**
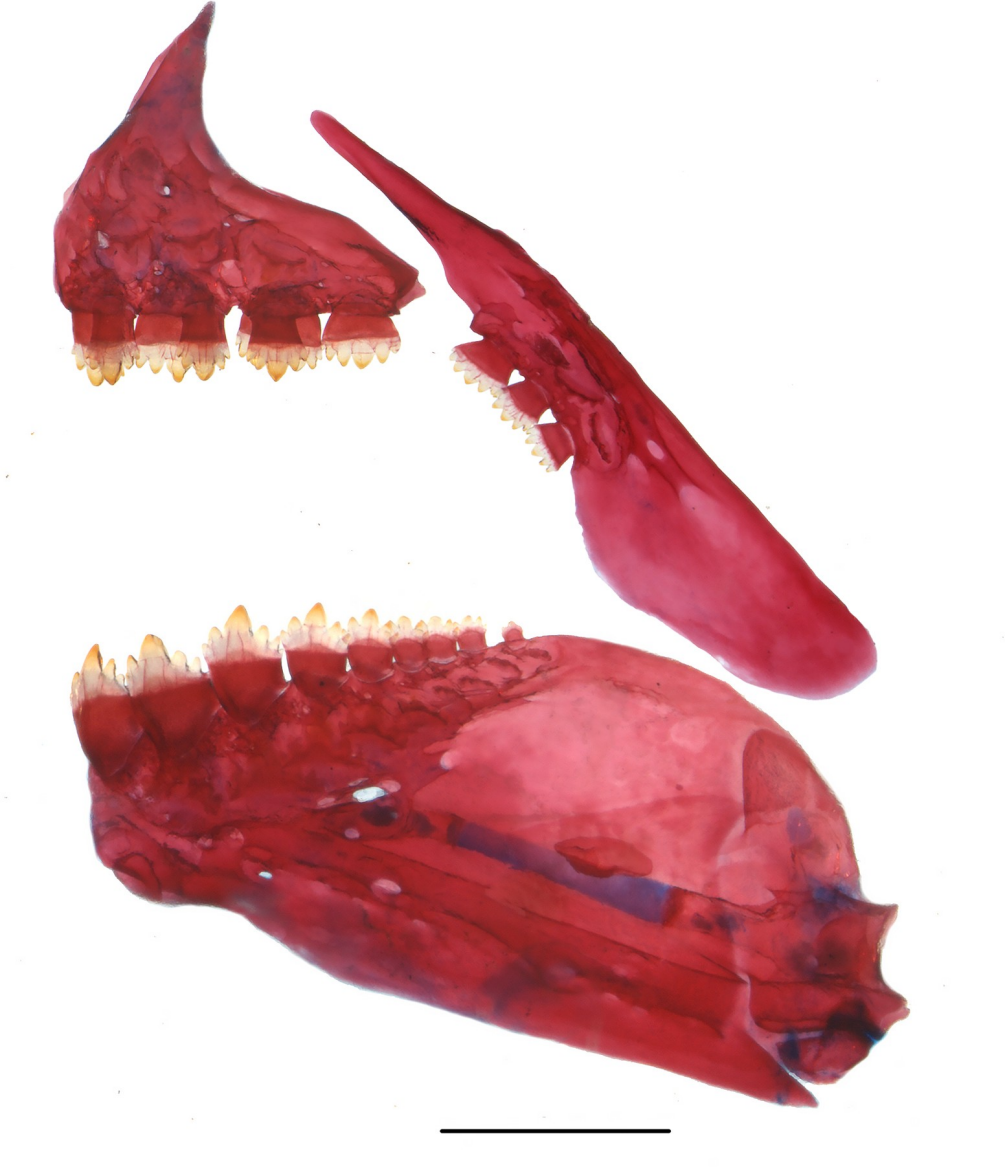
Upper and lower jaws of *Knodus nuptialis*. Lateral view of right side (image inverted), MZUSP 124828, paratype, 42.6 mm SL. Scale bar 1 mm.

**Fig 3:**
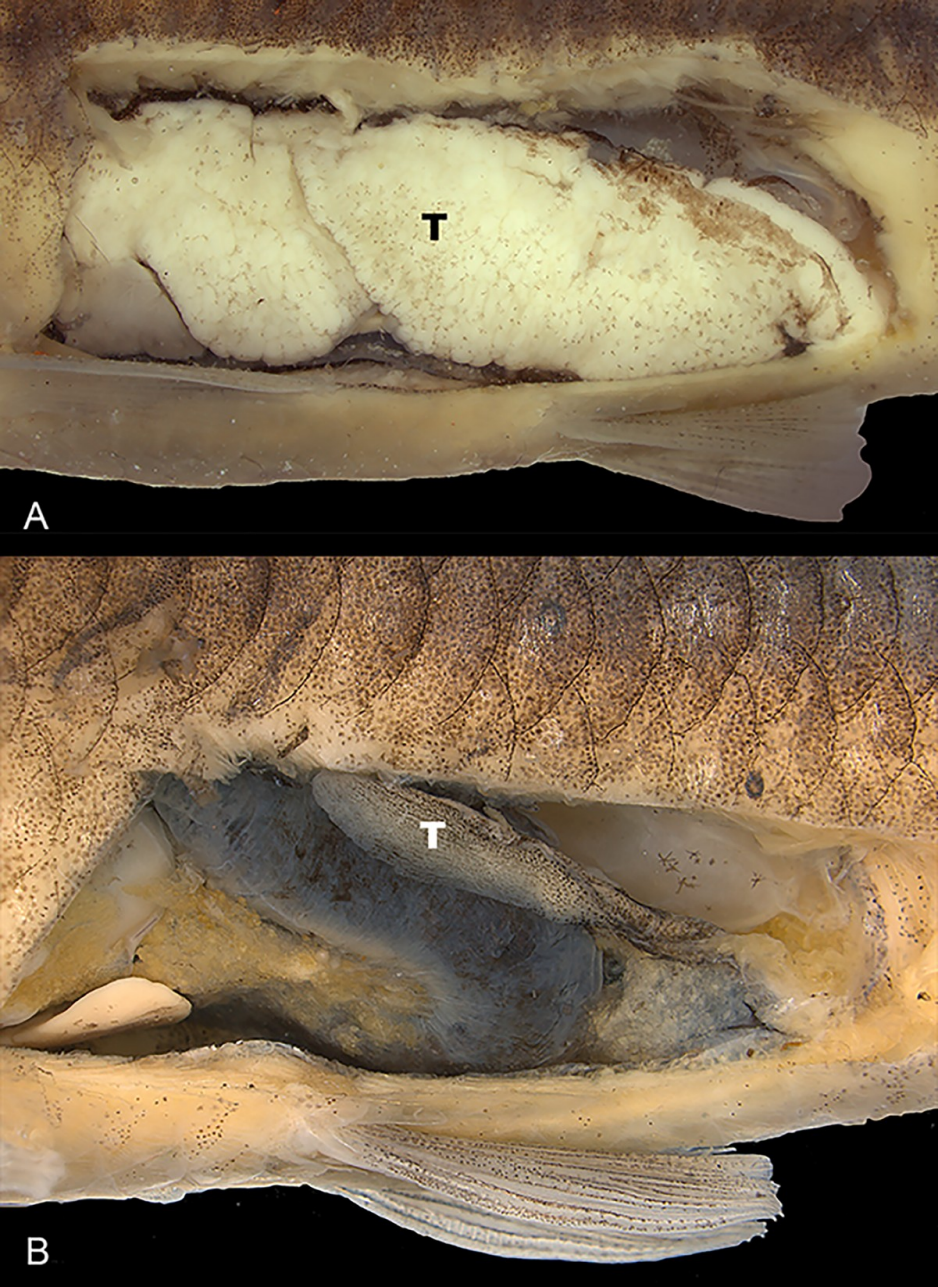
Dissected males of *Knodus nuptialis*. Lateral view of paratypes showing (A) extremely developed testicles of specimen collected in August (reproductive season), MZUSP 124828, 50.4 mm SL; (B) not fully-developed testicles specimen collected in October, MZUSP 97093, 61.5 mm SL.

**Fig 4:**
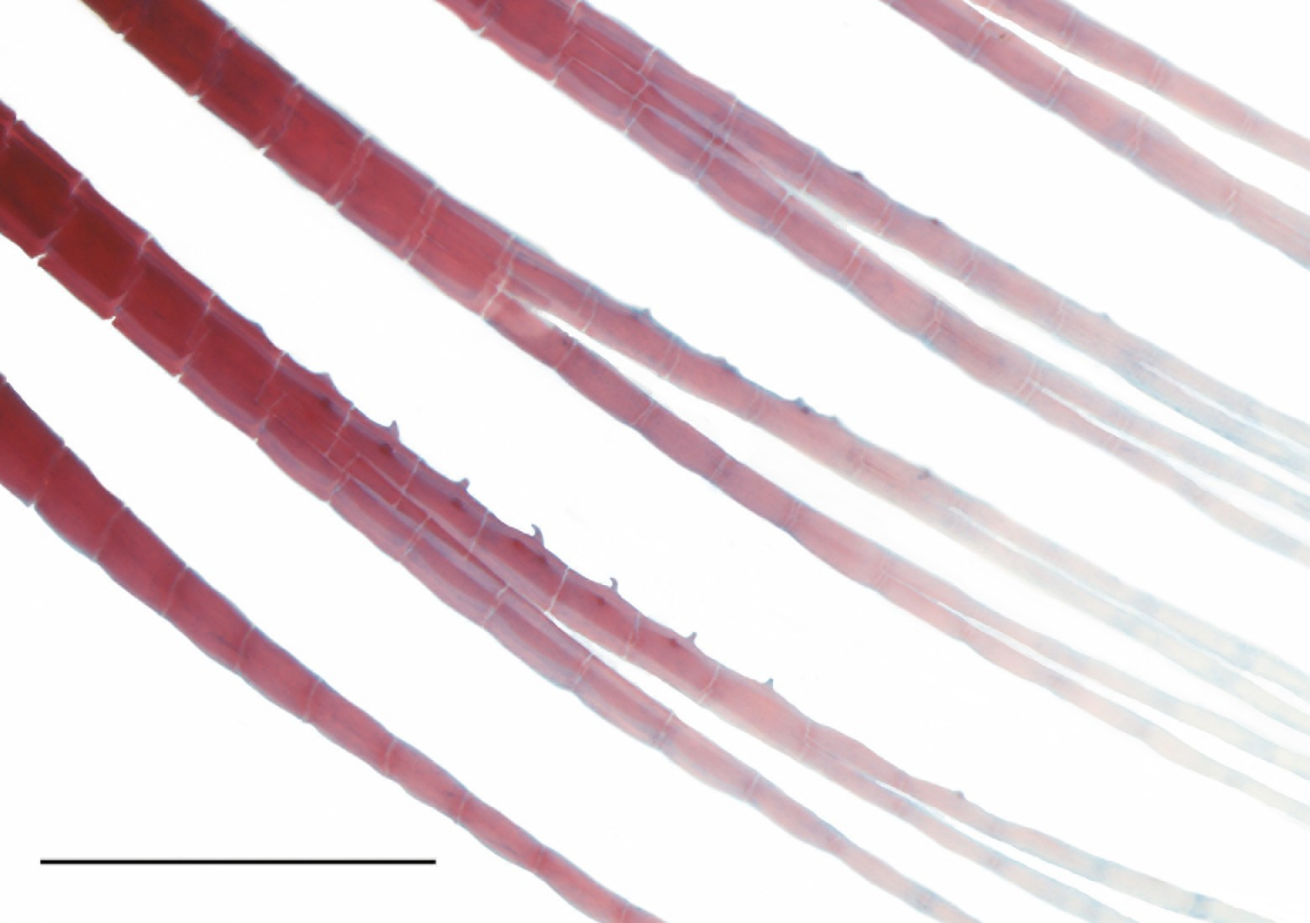
Anal-fin of *Knodus nuptialis* with bony hooks. First three branched anal-fin rays, MZUSP 124828, 50.2 mm SL, male paratype collected in the reproductive season.

**Fig 5:**
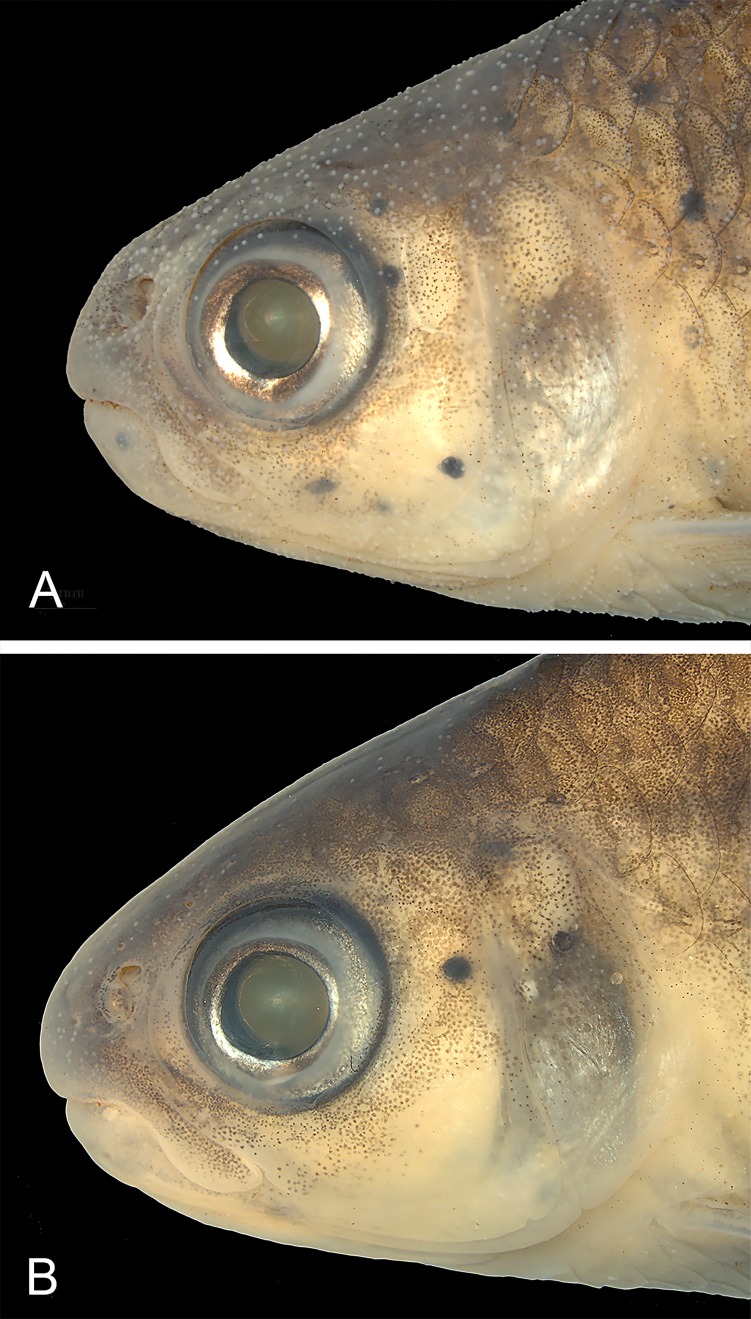
Head of *Knodus nuptialis* collected in the reproductive season. MZUSP 124828, paratypes, (A) male with dense concentration of breeding tubercles, 50.5 mm SL; (B) female with few, sparsely distributed breeding tubercles, 53.7 mm SL.

**Fig 6:**
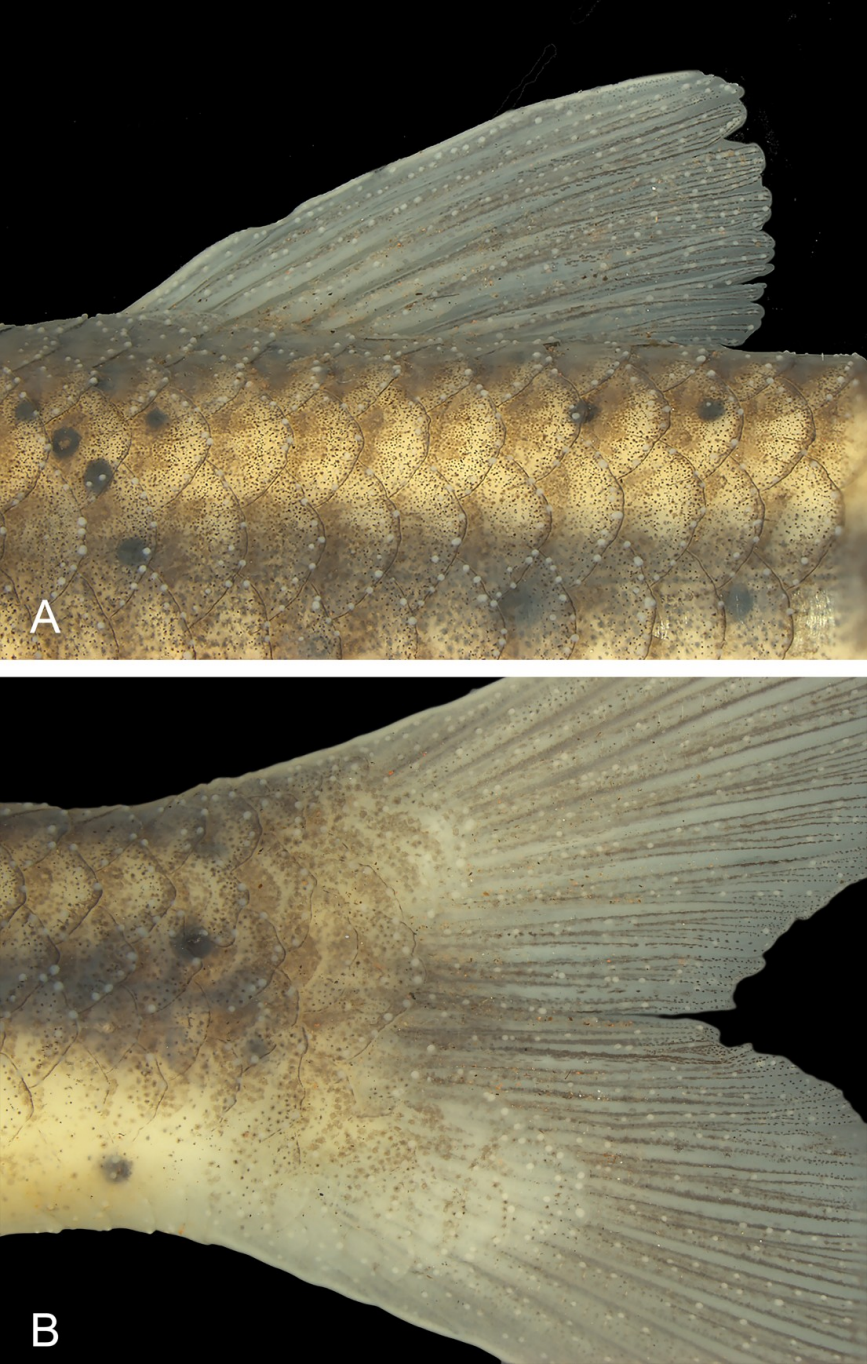
Distribution of breeding tubercles in *Knodus nuptialis*. MZUSP 124828, male paratype, 52.2 mm SL (A) dorsal scale rows and dorsal fin; (B) caudal fin.

**Fig 7:**
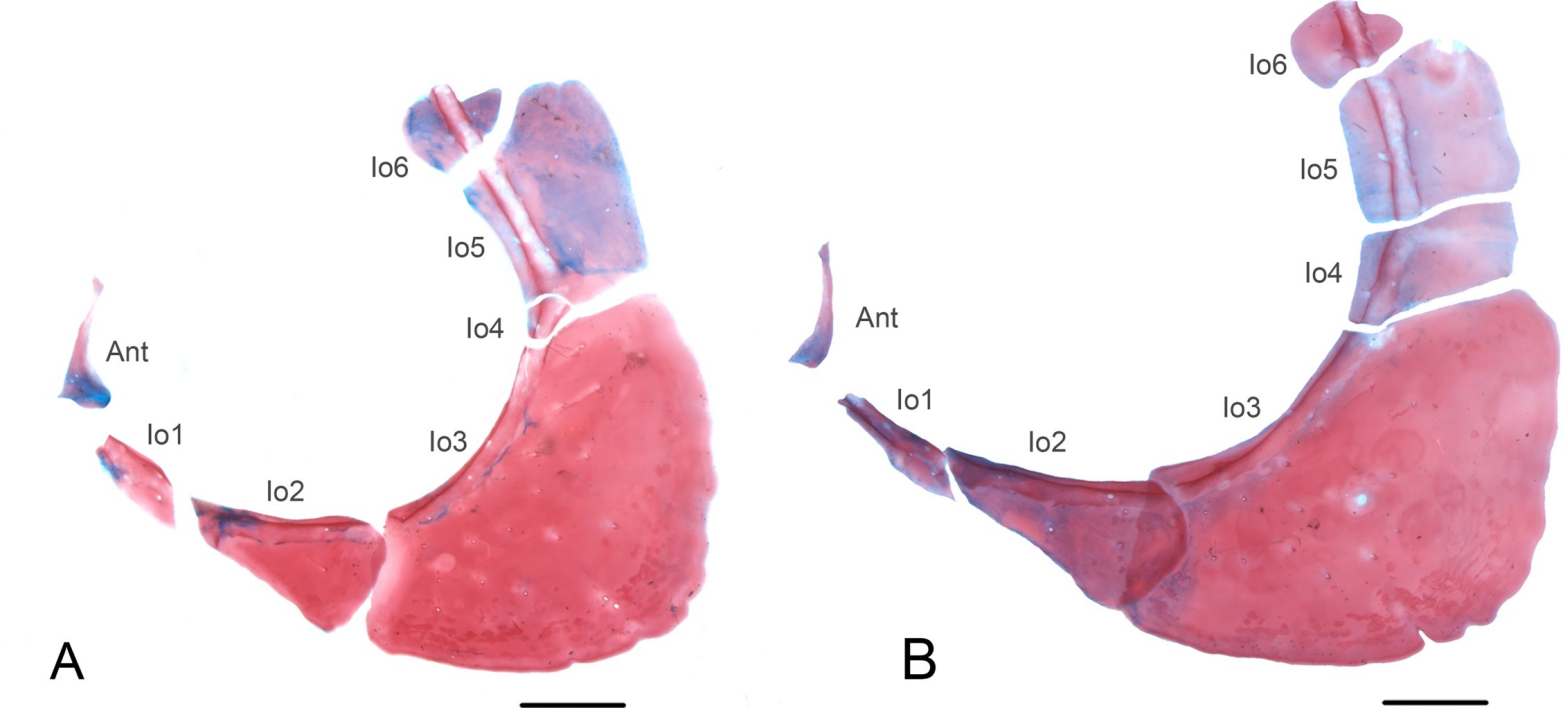
Circumorbital bones of paratypes of *Knodus nuptialis*. MZUSP 124828, showing variation in the form of infraorbital (Io) 4 (A) 42.6 mm SL; (B) 49.2 mm SL. Antorbital (Ant). Scale bar 1 mm.

**Fig 8:**
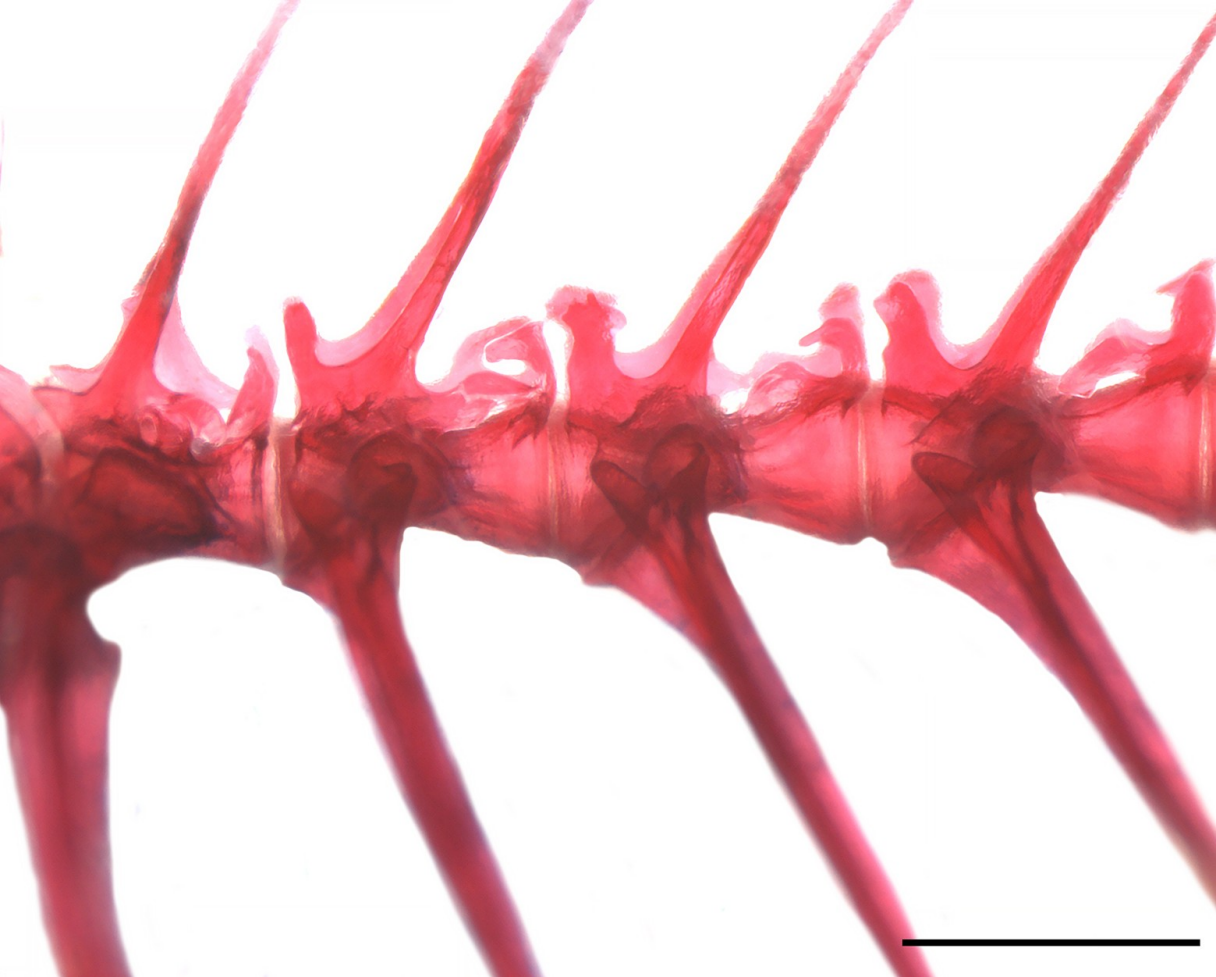
Vertebrae 5 to 8 of paratype of *Knodus nuptialis*. The form of pre- and postzygapophyses are shown, MZUSP 124828, 49.2 mm SL. Scale bar 1 mm.

**Table 1:** Morphometrics of *Knodus nuptialis*.

	Males					Females				
Characters	Holotype	n	Range	Mean	SD	n	Range	Mean	SD	*p* value
Standard length	46.5	74	27.5–59.0	44.3		73	22.0–73.0	42.7		
**Percents of Standard Length**										
Depth at dorsal-fin origin	26.2	74	24.2–29.3	26.5	1.1	73	22.6–32.3	27.0	1.5	0.376
Snout to dorsal-fin origin	53.8	74	52.3–58.7	55.0	1.2	73	52.7–62.2	56.1	1.6	0.365
Snout to pectoral-fin origin	22.1	74	20.0–26.6	23.1	1.1	73	20.9–28.6	24.1	1.6	0.401
Snout to pelvic-fin origin	48.8	74	45.0–55.0	49.0	1.7	73	46.6–54.5	50.2	1.7	0.405
Snout to anal-fin origin	65.1	74	64.3–70.8	67.1	1.3	73	65.1–72.7	68.5	1.6	0.396
Caudal peduncle depth	11.1	74	10.3–12.4	11.2	0.4	73	10.2–13.0	11.3	0.5	0.256
Caudal peduncle length	15.1	74	12.8–17.5	15.0	1.0	73	12.5–18.7	15.0	1.1	0.148
Pectoral-fin length	20.9	74	16.3–23.3	20.1	1.2	73	17.0–23.4	20.4	1.4	0.281
Pelvic-fin length	14.0	74	12.5–14.0	14.0	0.6	73	12.6–16.0	14.3	0.7	0.403
Dorsal-fin base length	11.6	74	09.5–12.7	11.4	0.7	73	10.2–14.1	11.5	0.7	0.337
Dorsal-fin height	21.0	74	18.2–23.6	20.5	1.1	73	18.3–23.6	21.1	1.1	0.462
Anal-fin base length	20.4	74	19.0–23.2	20.5	0.9	73	18.7–22.6	20.4	0.8	0.148
Anal-fin lobe length	15.1	74	13.0–17.3	15.1	1.0	73	14.0–20.4	16.1	1.1	0.159
Eye to dorsal-fin origin	41.8	74	38.7–44.8	41.5	1.0	73	39.6–45.4	42.4	1.0	0.361
Dorsal-fin origin to caudal-fin base	46.5	74	45.7–51.2	48.8	1.2	73	50.7–48.0	48.0	1.2	0.090
Head length	25.6	74	23.2–28.8	25.5	1.1	73	23.0–29.5	26.0	1.4	0.406
**Percents of Head Length**										
Horizontal eye diameter	41.0	74	34.7–43.7	38.7	1.8	73	34.5–42.7	38.6	2.0	0.280
Snout length	22.7	74	18.2–26.3	22.1	1.7	73	19.6–24.6	22.6	1.0	0.420
Least interorbital width	31.8	74	27.2–35.0	30.8	1.1	73	26.6–33.3	30.3	1.5	0.158
Upper-jaw length	36.3	74	36.3–44.4	40.5	1.7	73	36.1–43.4	39.1	1.7	0.079

n = number of specimens, SD = Standard deviation. Range does not include the holotype.

All specimens examined from Brazil, Pará, Altamira, Rio 13 de Maio at Small Hydroelectric Central Salto do Três de Maio, tributary of Rio Curuá, Rio Xingu basin, 8°44’59”S 55°1’58.8”W.

Holotype: MZUSP 124829, male, 46.5 mm SL, upstream dam, M. M. F. Marinho, P. Camelier, F. Dagosta, & V. Giovannetti, 7 August, 2015.

Paratypes: MZUSP 124828, 93 (22.0–56.0 mm SL, 5, C&S, 42.6–53.7 mm SL), INPA 58848 (7, 41.2–57.4 mm SL), MCP 54157 (7, 36.9–51.8 mm SL), MNRJ 51442 (7, 36.5–55.9 mm SL), collected with holotype. MZUSP 97093, 43 (26.0–73.0 mm SL, mol), upstream dam, J. Birindelli, L. Sousa, A. Netto-Ferreira, M. Sabaj-Perez & N. Lujan, 22 Oct 2007. MZUSP 101425, 18 (31.3–68.9 mm SL), upstream dam, Netto-Ferreira, A. L., Birindelli, J. L., Sousa, L. & Hollanda-Carvalho, P., 23 Jan 2009. MZUSP 124827, 59 (21.9–47.9 mm SL), downstream dam, O. T. Oyakawa, M. M. F. Marinho, P. Camelier & R. Burger, 12 Oct 2017.

### Diagnosis

*Knodus nuptialis* can be distinguished from all congeners, except *K*. *deuteronoides* Eigenmann in Eigenmann and *K*. *tiquiensis* Ferreira and Lima, by having the dentary teeth arranged in a continuous series, with teeth decreasing gradually in size posteriorly (*versus* arranged in a discontinuous series with the anterior teeth conspicuously larger, followed by abruptly smaller teeth posteriorly). *Knodus nuptialis* differs from *K*. *deuteronoides* by having 3–5 (rarely 3) premaxillary teeth in the outer row (*versus* 2–3 (rarely 3)), 4 scale rows below lateral line (*versus* 3), the origin of the dorsal fin closer to snout tip than to caudal-fin base (v*ersus* dorsal-fin origin in the middle of the distance between snout tip and caudal-fin base), the origin of the anal fin posterior to vertical crossing base of last dorsal-fin ray (*versus* anal-fin origin anterior to vertical crossing base of last dorsal-fin ray in *K*. *deuteronoides*; data from [13], and midlateral dark stripe reaching humeral spot (*versus* not reaching, humeral spot with a pale area behind; data from [14]. The new species can be distinguished from *K*. *tiquiensis* by having a single humeral spot (*versus* two) and the relatively narrow midlateral stripe (*versus* broad stripe). *Knodus nuptialis* can be further distinguished from all congeners, except *K*. *deuterodonoides*, *K*. *figueiredoi* Esguícero and Castro, *K*. *geryi* Lima, Britski and Machado, *K*. *meridae* Eigenmann, *K*. *orteguasae* Fowler, and *K*. *tiquiensis*, by having 12–15 branched anal-fin rays (*versus* 16–26). It can be further distinguished from *K*. *figueiredoi* by having inner premaxillary teeth with 5 to 8 cusps (*versus* 3), from *K*. *meridae* and *K*. *orteguasae* by having 4 scale rows between lateral line and pelvic-fin origin (*versus* 2 ou 3) and from *K*. *deuterodonoides* and *K*. *tiquiensis* by the features aforementioned. The presence of densely concentrated nuptial tubercles in mature males may also help to diagnose the new species.

### Description

Body comparatively small (largest examined specimen 73 mm SL). Head and body elongate and laterally compressed; greatest body depth at dorsal-fin origin. Profile distinctly convex from upper jaw to posterior nostril, slightly convex from latter point to dorsal-fin origin, straight along dorsal-fin base, slightly concave from latter point to adipose-fin origin, and concave from latter point to anteriormost dorsal-procurrent ray. Ventral body profile convex from tip of lower jaw to isthmus, nearly straight from that point to vertical through pectoral-fin origin, convex from latter point to pelvic-fin origin, and straight from that point to anal-fin origin. Ventral profile along anal-fin base straight and concave on caudal peduncle (Fig 1).

Mouth sub-terminal; lower jaw short, included in upper jaw when mouth closed. Posterior tip of maxilla reaching vertical through anterior border of pupil. Outer premaxillary tooth row with 3 (3), 4 (*138), or 5 (7) teeth, each with five cusps (5), inner row with 4 (*148) teeth with 5 to 8 cusps (5) (Fig 2). Maxilla with 2 (35) or 3 (*113) teeth, anterior larger teeth with 8 cusps, smaller posterior teeth with 3–5 cusps (5). Dentary with 6 (1), 7 (10), 8 (69), 9 (*59), or 10 (9) teeth, with 5 to 8 cusps, gradually decreasing in size posteriorly. First gill arch with 16 (1), 17 (*24), 18 (64), 19 (43), or 20 (12) rakers laterally positioned. Additional medial row of about 8 (2) tiny gill rakers. Branchiostegal rays 4 (5), 3 associated to anterior and 1 to posterior ceratohyal.

Scales cycloid, about 8 radii originating from focus of scale. Lateral line complete; perforated scales 35 (2), 36 (*38), 37 (68), 38 (28), or 39 (1). Predorsal scales 12 (23), 13 (101), or 14 (*23). In the predorsal scale row, the last scale has the posterior border notched where the base of the first unbranched dorsal-fin ray fits in. Scale rows between lateral line and dorsal-fin origin 4 (23) or 5 (*125); rows between lateral line and pelvic-fin origin 4 (*148); circumpeduncular scales 14 (*148). Single series of scales with sinuous posterior borders forming sheath along base of all anal-fin rays. Scales with sinuous posterior border covering proximal fourth of upper and proximal third of lower caudal-fin lobe.

Pectoral-fin rays i,8,i (7), i,9 (2), i,9,i (*97), i,10 (8), or i,10,i (28). Distal tip of longest pectoral-fin ray not reaching pelvic-fin origin. Pelvic-fin rays i,5,i (116), i,6 (20), or i,6,i (*12), tip of fin not reaching anal-fin origin. Dorsal-fin rays ii,7,i, (*143), or ii,8 (5). First dorsal-fin pterygiophore bifurcated proximally, its main portion inserting behind neural spine of 12^th^ (5) vertebral centrum. Distal margin of extended dorsal fin straight to slightly convex. Dorsal-fin origin closer to caudal-fin base than to snout tip. Base of last dorsal-fin ray situated slightly anterior to vertical through anal-fin origin. Supraneurals 7 (5), rod shaped, or with discrete enlargement of dorsal portion; last supraneural located anterior to spines of 11^th^ (4) or 12^th^ (1) vertebral centra. Anal-fin rays iii,12 (2), iii,13 (*28), iii,13,i (*2), iii,14 (81), or iii,15 (34), posteriormost ray adnate. Anal fin with short, anterior lobe including last unbranched ray plus first 5–6 branched rays. Distal margin of anal fin concave. First anal-fin pterygiophore inserting behind haemal arch of centra 19^th^ (2) or 20^th^ (3). Adipose fin present. Principal caudal-fin rays i,9,8,i (*31). Dorsal procurrent rays 11 (4) or 12 (1), ventral procurrent rays 10 (1), 11 (2), or 12 (2). Total vertebrae 36 (2) or 37 (3).

### Color in alcohol

Ground color pale to yellowish brown (Fig 1A). Upper part of head dark from tip of snout to end of supraoccipital spine, dark color continuing back over predorsal scales; minute melanophores around eye, extending laterally over maxilla, first, second, fourth, fifth and sixth infraorbitals, upper half of third infraorbital, upper half of interopercle and opercle; anterior part of lower jaw with scattered melanophores. Melanophores spread all over upper part of trunk, those on central part of scales smaller and heavily concentrated, turning this area darker than scale border, where chromatophores are larger and sparsely distributed. Scattered melanophores on lower part of body below lateral line, more numerous above anal-fin base. Vertically elongate humeral spot occupying 3 to 4 longitudinal scale rows vertically and becoming narrower downward. Midlateral dark stripe from upper portion of opercle to caudal-fin base, slightly enlarged over caudal peduncle, and fading toward tip of middle caudal-fin rays. All fins hyaline with scattered melanophores on dorsal, adipose, caudal and anal fins; very few chromatophores on pectoral, and pelvic fins.

### Color in life

Dorsal portion of body from head to caudal peduncle with yellow ground coloration, darker than ventral portion (Fig 1B). Ventral portion of body below midlateral stripe clear, with brilliant white or silver coloration. Dorsal portion of eye yellow, ventral portion grey. Infraorbitals 3–6 and opercular areas light rose. Midlateral stripe with brilliant silvery coloration as result of guanine concentration. Proximal portion of dorsal, adipose, pectoral, pelvic fins, and caudal-fin lobes yellow. Proximal portion of anal-fin rays light red. Distal portion of unbranched and first branched dorsal, pelvic and anal-fin rays milky white.

### Sexual dimorphism and reproductive traits

Samples of the new species are represented by specimens collected in the same area in three distinct periods (beginning of August, MZUSP 124829 and 124828, October, MZUSP 97093 and 124827, and January MZUSP 101425). All males collected in August with relatively wide range of sizes (32.4–57.8 mm SL), present extremely developed gonads, occupying a large portion of the abdominal cavity (Fig 3A). Large females (more than 54.0 mm SL) collected in August with fully developed ovocytes, smaller ones immature or having their gonads in maturation. Males and females collected in October and January are adults not in the reproductive stage, considering the development of their gonads, or are immatures (21.9–73.0 mm SL) (Fig 3B). It characterizes a seasonal pattern of reproductive dynamics, found in most other Characiformes. All sexual dimorphic traits of *Knodus nuptialis* were observed only in mature specimens (those collected in August), described below.

Males with gill gland on the anterior portion of the lower branch (hypobranchial + ceratobranchial) of first gill arch, encompassing about 7–10 gill filaments. This feature is useful to sex *K*. *nuptialis* in the reproductive season (August), since it is developed in all the mature males analyzed (32.4–57.8 mm SL). Females lack the gill gland.

Tiny bony hooks on the anal fin present in mature males larger than 41.3 mm SL (n = 24) (Fig 4); smaller mature males (32.4–48.8 mm SL, n = 39) without hooks. Hooks always associated with a thick, probably glandular mass of tissue. Hooks restricted to the middle portions of first branched anal-fin ray (n = 5), of first two branched rays (n = 10), first three branched rays (n = 2), first four branched rays (n = 1), last unbranched and first branched ray (n = 1) and of last unbranched and first two branched rays (n = 2), always at the posterior branch of those rays. Hooks distribution is not correlated to size. Hooks absent on pelvic-fin rays. Females lack bony hooks in all fins.

All mature males with nuptial tubercles distributed all over head and body, less concentrated or absent in the ventral region between gular area and pelvic fins. Nuptial tubercles highly concentrated in the dorsal portion of head (Fig 5A). In the scaled portion of body, nuptial tubercles are commonly arranged in the distal border of the scales (Fig 6A). On fins, tubercles are common along the lepidotrichia (Fig 6B). Large females (more than 55.0 mm SL) with nuptial tubercles sparsely distributed on dorsal and lateral portions of head (Fig 5B), absent on all fins. Smaller females lacking tubercles. Females (73 specimens, SL 25–73 mm) grow bigger than males (75 specimens, SL 26–58 mm). Other measurements are not sexually dimorphic (Table 1).

### Etymology

The species name *nuptialis* is from the Latin meaning pertaining to marriage, in allusion to the presence of a series of sexual dimorphic traits (hooks, gill glands and nuptial tubercles) during the breeding season of this species.

### Distribution

**Fig 9:**
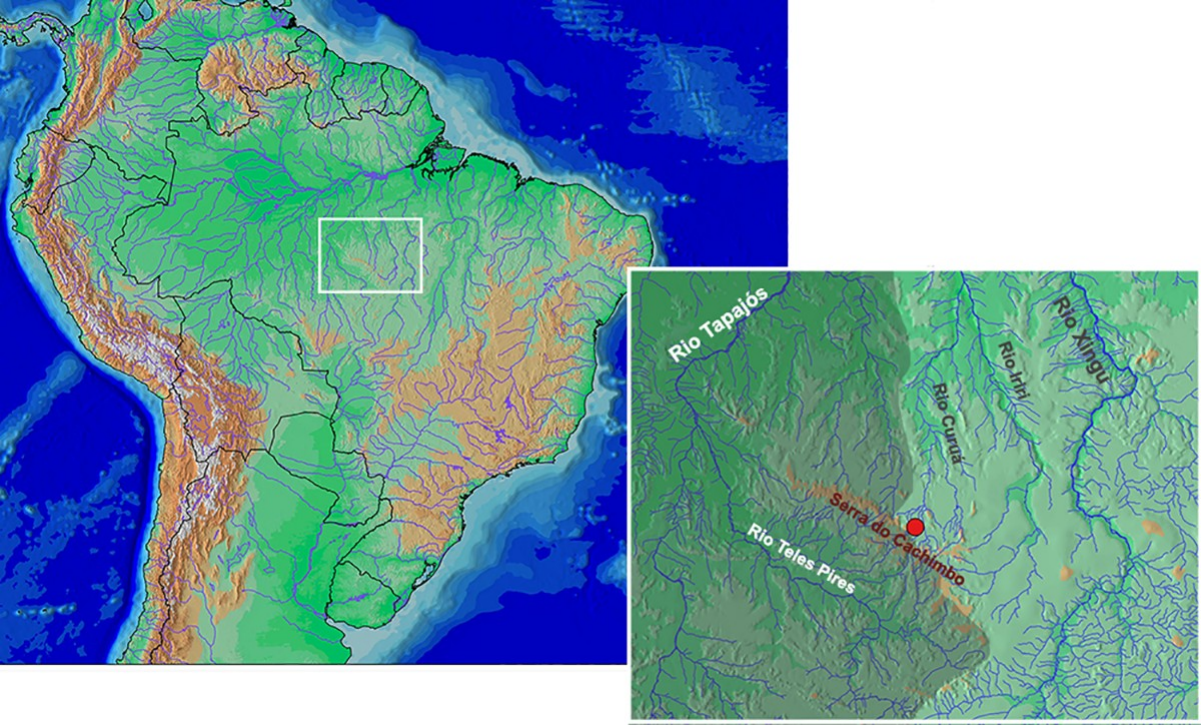
Geographic distribution of *Knodus nuptialis*. Rio 13 de Maio, tributary of Rio Curuá, upper Rio Xingu basin at Serra do Cachimbo, state of Pará, Brazil. Shaded area corresponds to rio Tapajós basin.

**Fig 10:**
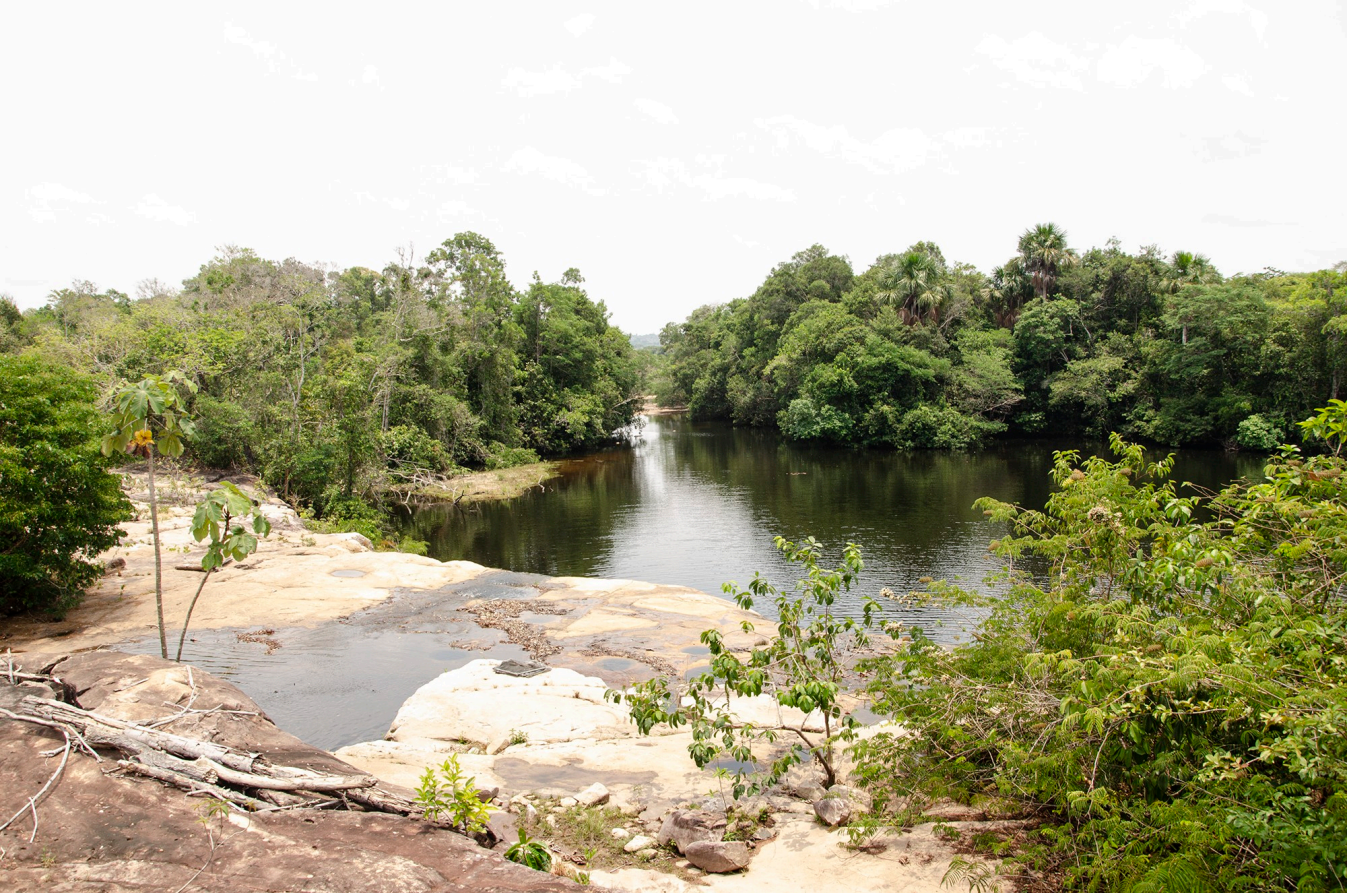
Type locality of *Knodus nuptialis*. Rio 13 de Maio at 8°44’59”S 55°1’58.8”W, tributary of Rio Curuá, upper Rio Xingu basin at Serra do Cachimbo.

*Knodus nuptialis* is so far known from the Rio 13 de Maio, tributary of Rio Curuá, upper Rio Xingu basin in the state of Pará, Brazil (Figs 9 and 10).

## Discussion

*Knodus nuptialis* share the traditional morphological features that define the genus, especially the possession of scales on the caudal fin, sometimes slightly elongate extending to about two thirds over base of caudal-fin rays. Therefore, the new species is here included in *Knodus*. It is noteworthy, however, that *K*. *nuptialis* shares some similarities with *Myxiops aphos* Zanata and Akama.

Zanata and Akama [15] listed seven features present in *Myxiops aphos*, unusual in other characids, what led the authors to propose a new genus. Five of these are also present in *K*. *nuptialis*: (1) fusion of infraorbitals in variable fashions, reducing the number of elements surrounding the eyes. Among the five cleared and stained specimens of *K*. *nuptialis*, there is a considerable variation in the form of infraorbitals, especially infraorbitals 4 and 5 (see Fig 7), and in one specimen infraorbital 5 and 6 are fused (or infraorbital 6 may be missing). (2) possession of a cheirodontin-like teeth (somewhat pedunculated, expanded and compressed distally). *Knodus nuptialis* has expanded teeth, compressed distally, but not as pedunculated as in *M*. *aphos*. (3) maxillary teeth forming a continuous series with the premaxillary teeth. (4) possession of elaborated pre- and postzygapophyses (Fig 8) and (6) possession of nuptial tubercles. Zanata and Akama [15] did not consider the protuberances over the body of *M*. *aphos* as nuptial tubercles, but it is demonstrated below that it is indeed a sexual dimorphic trait of mature males, comparable to those observed in *K*. *nuptialis*. Therefore, of the seven features present in *M*. *aphos*, only two are absent in *K*. *nuptialis*, (2) a single premaxillary tooth row and (7) base of anal fin without scales covering basal portion of anal-fin rays. All those features, however, need to be tested in a phylogenetic framework within the Characidae to evaluate whether they are indicative of a close relationship between *M*. *aphos* and *K*. *nuptialis*. Given the lack of a phylogenetic diagnosis for most genera in the Characidae, including *Knodus* and *Myxiops*, we take a conservative position and describe the new species in *Knodus*, following the traditional definition of the genus.

### Comments on nuptial tubercles and gill gland in characiforms

Nuptial tubercles, also referred to as breeding tubercles, are protuberances formed by intense proliferation of epidermal cells which, in the case of *Knodus nuptialis*, are characterized by small white dots widespread over the head, scales and fins (in males) of mature specimens. Wiley and Collette [16] reported the presence of breeding tubercles in several fish families currently assigned to the orders Characiformes, Cypriniformes, Gonorynchiformes, Osmeriformes, Perciformes, and Salmoniformes. Within the Characiformes, they have been described in the Characidae [16], Distichodontidae [17], Lebiasinidae, and Parodontidae [16]. The function of such structures is still unclear, but it may facilitate the contact between individuals during courtship and spawning [16].

Comparisons of breeding tubercle morphology and histology revealed significant differences among taxa [16]. There are two generalized types of tubercles, both present in the Characiformes: 1) those formed by keratinized cells organized to form a distal cap, found in species of Parodontidae [16] and *Creagrutus guanes* Torres-Mejia and Vari [18] and 2) those formed by proliferation of hypertrophied epithelial cells with no keratinization, found in the Pyrrhulininae [16] and possibly *Mixyops aphos* [15] and *Knodus nuptialis*. All are characterized by small, white dots scattered on body.

Several authors after Wiley & Collette [16] have pointed out the presence of breeding tubercles as part of the description of new species, but this information remained diffuse in the literature. For the purpose of providing a starting point for future studies in the area, we update the list of characiform species presenting such epidermal protuberances (Table 2). Nine species and two genera of the Characidae (*Deuterodon* and *Bryconacidnus*) are for the first time reported to have breeding tubercles: *Astyanax* (4), *Deuterodon* (1), *Bryconacidnus* (2), *Bryconamericus* (1) and *Knodus* (1). A total of 59 species of Characiformes are known to present breeding tubercles.

**Table 2:** Species of Characiformes with breeding tubercles.

Taxon	Sex distribution	Reference
**DISTICHODONTIDAE**		
*Nannocharax reidi*	-	[17]
*Nannocharax rubrolabiatus*	-	[17]
**PARODONTIDAE**		
*Apareiodon affinis*	-	[16]
*Apareiodon machrisi*	Only males	[19]
*Apareiodon vladii*	Both sexes	[20]
*Parodon alfonsoi*	Only males	[32]
*Parodon apolinari*	Both sexes	[22, 16]
*Parodon atratoensis*		[21]
*Parodon buckleyi*	-	[16]
*Parodon caliensis*	Only males	[21]
*Parodon carrikeri*	Only males	[23]
*Parodon hilarii*	-	[16]
*Parodon magdalenensis*	Only males	[16]
*Parodon moreirai*	-	[24]
*Parodon nasus*	-	[16]
*Parodon pongoensis*	-	[16]
*Parodon suborbitalis*	-	[16]
*Saccodon dariensis*	-	[16]
*Saccodon wagneri*	-	[16]
**LEBIASINIDAE**		
**Lebiasininae**		
*Lebiasina ardilai*	-	[16]
*Lebiasina melanoguttata*	Only males	[27]
*Lebiasina yepezi*	Only males	[26]
**Pyrrhulininae**		
*Copeina* (probably *Copella*) sp.	Only males	[26]
*Copella compta*		[25]
*Nannostomus beckfordi*	-	[27]
*Nannostomus bifasciatus*	Only males	[16]
*Nannostomus limatus*	-	[27]
*Nannostomus unifasciatus*	Only males	[16]
*Nannostomus marginatus*	-	[27]
*Nannostomus nitidus*	-	[27]
*Pyrrhulina* sp.	Only males	[16]
**CHARACIDAE**		
**Stethaprioninae** (*sensu* Mirande, 2018)		
*Asyanax aeneus*	Only males	[23]
*Astyanax aramburui*	Only males	[23]
*Astyanax epiagos*	-	[28]
*Astyanax eremus*	-	[29]
*Astyanax guaricana*		Present paper (MZUSP 112225)
*Astyanax gymnodontus*	-	[30]
*Astyanax jenynsii*	-	Present paper (MZUSP 110321)
*Astyanax jornadensis*	-	Present paper (MZUSP 99137)
*Astyanax lorien*	Both sexes	[31]
*Astyanax ojiara*	-	[30]
*Astyanax parahybae*	-	[30]
*Astyanax rupestris*	Both sexes	[31]
*Astyanax scabripinnis*	Both sexes	Present paper (MZUSP 45927)
*Astyanax troya*	-	[20]
*Astyanax* sp. (*A*. *scabripinnis* complex)	-	[32]
*Deuterodon iguape*	Only males	Present paper (MZUSP 114835)
*Moenkhausia comma*	Only males	[33]
*Myxiops aphos*	Only males	[15], present paper
**Stervardiinae**		
*Bryconacidnus ellisi*	-	Present paper (MZUSP 121041)
*Bryconacidnus hemigrammus*	-	Present paper (MZUSP 12060)
*Bryconamericus microcephalus*	Only males	Present paper (MZUSP 80012)
*Bryconamericus emperador*	Only males	[34]
*Bryconamericus thomasi*	Only males	[23]
*Bryconamericus* sp. (from Pamaná)	Only males	[35]
*Ceratobranchia binghami*	Only males	[36]
*Creagrutus guanes*	Both sexes	[18]
*Knodus nuptialis*	Both sexes	Present paper
*Knodus* sp. (from rio Madeira basin)	Only males	[37]

Based on the available information, the presence of breeding tubercles is more frequently found exclusively in males, but it may occur in both sexes in several species (Table 2). Furthermore, breeding tubercles are mostly found in mature specimens [16], possibly only well developed for a restricted period of time during the height of the breeding season [17]. In *Knodus nuptialis*, breeding tubercles are present in both males and females, strictly in mature specimens, when the gonads are fully developed (Fig 3A). Zanata and Akama [15] mentioned a distinct scenario for *Myxiops aphos*: “in *Myxiops* the accumulation of epithelial cells is more conspicuous in juveniles and occur both in males and females”, then justifying why the term breeding tubercle was not used. We analyzed the holotype and paratypes of *Myxiops aphos* deposited at MZUSP (18 specimens) and sexed eight specimens through a small incision in the abdominal cavity. Four smaller (40.2–47.2 mm SL) specimens, widely covered by breeding tubercles, proved to be males, whereas four larger ones (46.5–56.6 mm SL) lacking tubercles are females. Contrasting to the statement in [15] that breeding tubercles are more conspicuous in juveniles, and “sometimes nearly absent in larger specimens”, the smaller specimens are fully mature males, and the larger ones are mature females without tubercles. Therefore, breeding tubercles is considered a sexual dimorphic character in *M*. *aphos*. The gonads of the holotype of *M*. *aphos* was not examined but, considering it is a large specimen (56.0 mm SL) lacking breeding tubercles, and that it was collected along with the mature paratypes, it is most probably a female.

The distribution of breeding tubercles on the body may vary intraspecificaly (see Sexual dimorphism and reproductive traits of *K*. *nuptialis*) and among taxa. Based on the available information, there are slightly distinct patterns of tubercle distribution among characiform taxa (literature citations listed in Table 2). In the Distichodontidae, they are distributed on the dorsal and lateral portions of the head, distal margins of body scales and along the rays of all fins. In the Parodontidae, the common pattern of distribution is over the dorsal and lateral portions of head. Within the Lebiasinidae, there seems to have three distinct patterns: solely at the posterior margins of scales in the species of *Copella* and *Pyrrhulina*, at the ventral portion of the head in *Nannostomus*, and at pectoral, pelvic, and caudal fins, and posterior margins of body scales in *Lebiasina*. In the Characidae, the common pattern of distribution is on the dorsal and lateral portions of the head and the posterior margin of body scales. The exceptions are mature males of *Bryconamericus microcephalus*, *Knodus nuptialis*, and *Deuterodon iguape*, which further possess breeding tubercles along the lepdotrichia of all fins (similar to their distribution in the Distichodontidae).

Another interesting feature observed in *Knodus nuptialis* is the presence of a gill gland in mature males. The gill gland has a widespread occurrence in characiform fishes and was reported in male specimens of many groups [38]. The development, contents, nature and phylogenetic significance of the gland are presented in [39]. The substance hypothesized to be a pheromone produced by mature males during the reproductive season is apparently released to stimulate the females during courtship [40], and development of gill glands is correlated with testis maturation [41].

According the developmental pattern of sexually dimorphic features described in the sexual dimorphism section, it seems quite evident that in *Knodus nuptialis* the gland, nuptial tubercles and bony hooks are found fully developed during the reproductive season but are absent in males of all sizes (29.9–61.3 mm SL) collected two months afterwards. This indicates that these features are reabsorbed or lost after spawning. Therefore, such sexual dimorphic trait in *K*. *nuptialis* are useful and functional only in males during the reproductive season and after this period, they become atrophied, and eventually disappears.

The function of gill glands, breeding tubercles, and bony hooks are still unknown but they are related to reproductive behavior. The morphological seasonality of males presenting similar sexual dimorphism in the Characiformes need to be further investigated, as they can provide useful information for studies of systematics and evolution of the order, as well as for studies of reproductive biology, which can help to establish conservation policies for the species of Characiformes.

## Comparative material examined

*Astyanax guaricana*: MZUSP 112225, 10 paratypes, Rio Cubatão, Paraná, Brazil *Astyanax jenynsii*: MZUSP 110321, 6, Rio Paraíba do Sul, São Paulo, Brazil *Astyanax jordanensis*: MZUSP 99137, holotype, Rio Iguaçu basin, Paraná, Brazil *Astyanax scabripinnis*: MZUSP 45927, Rio Paraíba do Sul, São Paulo, Brazil. *Bryconacidnus ellisi*: MZUSP 121041, 8, Rio Madre de Dios basin, Peru. *Bryconacidnus hemigrammus*: MZUSP 121060, 20, lower Rio Urubamba, Cuzco, Peru. *Bryconamericus microcephalus*: MZUSP 80012, 40, Rio Ribeira de Iguape, São Paulo, Brazil. *Deuterodon iguape*: MZUSP 114835, Rio Ubatubinha, costal drainage, São Paulo, Brazil. *Knodus geryi*: MZUSP 78863, 28 paratypes, upper Rio Paraguai, Mato Grosso, Brazil. *Knodus tiquiensis*: MZUSP 81166, 1 paratype, MZUSP 85086, 1 paratype, MZUSP 85042, 3, Rio Negro basin, Brazil. *Myxiops aphos*: MZUSP 81026, holotype, MZUSP 81025, 17 paratypes, 4 c&s, tributary of Rio Paraguaçu, Lençóis, Bahia, Brazil.
